# Ceoliac disease

**DOI:** 10.1186/2045-7022-1-S1-S33

**Published:** 2011-08-12

**Authors:** Riccardo Troncone

**Affiliations:** 1 University Federico II, Department of Pediatrics & European Laboratory for the Investigation of Food-Induced Diseases, Naples, Italy

## 

Celiac disease is a T-cell mediated chronic inflammatory disorder with an autoimmune component. Altered processing by intraluminal enzymes, changes in intestinal permeability, and activation of innate immunity mechanisms seems to precede the activation of the adaptive response. Significant progress has been made in the understanding of the cellular and molecular basis of CD and in the consequent identification of potential targets for therapy. Recently, it has been shown that gliadin peptides are highly resistant to digestive processing by pancreatic and brush border proteases. Enzyme supplement therapy using bacterial prolyl endopeptidases has been proposed to destroy T-cell multipotent epitopes. The identification of T-cell stimulatory gliadin sequences is important. Breeding programs and/or transgenic technology may lead to production of wheat that is devoid of biologically active peptide sequences. The identification of specific epitopes may also provide a target for immunomodulation of antigenic peptides. Other promising areas include preventing gliadin presentation to T cells by blocking HLA binding sites, use of tTG inhibitors, and assessing IL-10 as a tool for promoting tolerance. However, evidence that gluten toxicity is not dependent only on T-cell recognition is growing; activation of innate immunity has been demonstrated and antibodies to IL-15 proposed, particularly in refractory sprue because of the IEL activating role of IL-15. However, one should realize that CD is a benign disease and dietary treatment is safe, although strenuous An immunomodulatory approach will need to have a safety profile equivalent to that of the GFD, but with the advantage of increased compliance. Another area of important changes for CD concerns the diagnostic protocol. In 1990 ESPGHAN has revised its former diagnostic criteria laid down in 1970. The two requirements mandatory for the diagnosis of celiac disease (CD) remain: 1) the finding of villous atrophy with hyperplasia of the crypts and abnormal surface epithelium, while the patient is eating adequate amounts of gluten; and 2) a full clinical remission after withdrawal of gluten from the diet. However, important changes that might have an impact on the diagnostic procedures for CD, have occurred in recent years. Tests based on the detection of anti-endomysium antibodies (EMA), and subsequently of anti-tTG, have been increasingly used as an initial screen for CD. Serological tests are largely responsible for the recognition that CD is not a rare disease; moreover, with the notion of the relatively high prevalence of CD has become increasingly recognised its broad spectrum of clinical presentations. The growing contribution of serology, together with the recognition of a wider spectrum of histological changes (see below), and the contribution by genetic tests, demonstrate the necessity to move on to a revised diagnostic approach, but until serological methods are improved, the genetic make up of celiac patients is better defined, it seems wise for a diagnosis of celiac disease still rely on a combined approach based of clinical criteria, histology, serology and genetics.

